# Fiberoptic hemodynamic spectroscopy reveals abnormal cerebrovascular reactivity in a freely moving mouse model of Alzheimer’s disease

**DOI:** 10.3389/fnmol.2023.1163447

**Published:** 2023-07-03

**Authors:** Daniel S. Gareau, Nicholas RochaKim, Arnab Choudhury, Michael Bamkole, Matija Snuderl, Julia Zou, Anna Yaroslavsky, Steven L. Jacques, Sidney Strickland, James G. Krueger, Hyung Jin Ahn

**Affiliations:** ^1^Laboratory of Investigative Dermatology, The Rockefeller University, New York, NY, United States; ^2^Department of Pharmacology, Physiology and Neuroscience, Rutgers-New Jersey Medical School, Newark, NJ, United States; ^3^Department of Pathology, NYU Langone Health and Grossman School of Medicine, New York, NY, United States; ^4^Department of Physics and Applied Physics, University of Massachusetts, Lowell, MA, United States; ^5^Department of Bioengineering, University of Washington, Seattle, WA, United States; ^6^Laboratory of Neurobiology and Genetics, The Rockefeller University, New York, NY, United States; ^7^Brain Health Institute, Rutgers University, Piscataway, NJ, United States

**Keywords:** Alzheimer’s disease, cerebrovascular reactivity, fiberoptic spectroscopy, cerebral blood volume, hypercapnia

## Abstract

Many Alzheimer’s disease (AD) patients suffer from altered cerebral blood flow and damaged cerebral vasculature. Cerebrovascular dysfunction could play an important role in this disease. However, the mechanism underlying a vascular contribution in AD is still unclear. Cerebrovascular reactivity (CVR) is a critical mechanism that maintains cerebral blood flow and brain homeostasis. Most current methods to analyze CVR require anesthesia which is known to hamper the investigation of molecular mechanisms underlying CVR. We therefore combined spectroscopy, spectral analysis software, and an implantable device to measure cerebral blood volume fraction (*CBVF*) and oxygen saturation (*S_O2_*) in unanesthetized, freely-moving mice. Then, we analyzed basal *CBVF* and *S_O2,_* and CVR of 5-month-old C57BL/6 mice during hypercapnia as well as during basic behavior such as grooming, walking and running. Moreover, we analyzed the CVR of freely-moving AD mice and their wildtype (WT) littermates during hypercapnia and could find impaired CVR in AD mice compared to WT littermates. Our results suggest that this optomechanical approach to reproducibly getting light into the brain enabled us to successfully measure CVR in unanesthetized freely-moving mice and to find impaired CVR in a mouse model of AD.

## 1. Introduction

Cerebral perfusion is tightly regulated in response to ischemia or metabolic demand, and proper regulation of cerebral perfusion is therefore a fundamental property of cerebral blood vessels. Cerebrovascular reactivity (CVR) is a primary mechanism that enables adjustment of cerebral blood flow to maintain proper metabolic supply for brain tissues and is coordinated by the interaction of neurons, glia, endothelium, smooth muscle cells, and pericytes (Girouard and Iadecola, 2006). The partial pressure of arterial carbon dioxide (PaCO_2_) is the most potent vascular factor which can induce CVR (Perry et al., 2014). Increased PaCO_2_ (hypercapnia) causes dilation of cerebral arteries and arterioles and leads to an increase in cerebral blood flow (CBF). Impaired CVR has been implicated in Alzheimer’s disease (AD) (Iadecola, 2004; Hardee and Zagzag, 2012). AD patients show decreased cerebral hemoglobin oxygen saturation (*S_O2_*) during verbal fluency tasks (Hock et al., 1997) and a failure of CBF increase during visual stimulus tests (Mentis et al., 1996). Impaired CO_2_ vasoreactivity is associated with cognitive deficits in hypertension patients (Hajjar et al., 2014), and blood oxygenation level-dependent (BOLD) functional Magnetic Resonance Imaging (fMRI) study showed impaired CVR to hypercapnia in patients with AD and amnestic mild cognitive impairment (MCI) patients (Cantin et al., 2011). However, several studies also showed that CVR to hypercapnia did not differ between AD and age-matched healthy control subjects (Kuwabara et al., 1992; Jagust et al., 1997; Rodell et al., 2012). Furthermore, a recent study using a mouse model of AD, J20-hAPP, showed that blood volume in the brains of AD mice under hyperoxia was substantially enhanced compared to wildtype (WT) controls (Shabir et al., 2020). Therefore, more studies are required to understand the mechanism of the impairment of CVR in AD.

Several diagnostic modalities, including fMRI (Stobart et al., 2023), and laser Doppler flowmetry (LDF; Park et al., 2014; Munting et al., 2021) have been used to measure vascular reactivity in animal studies and patients. In animal studies, these techniques require anesthesia in order to minimize movement artifacts, which prevents analysis of cerebral perfusion during the animals’ movement. In addition, most anesthetic agents including isoflurane and ketamine, are known to significantly modulate or inhibit vascular smooth muscle cell function (Akata et al., 2001, 2003; Akata, 2007; Masamoto and Kanno, 2012), leading to modified cerebrovascular function. Compared to fMRI, fiberoptic spectroscopy is inexpensive and portable and offers a higher temporal resolution. While the Near-Infrared light (650–950 nm) used in fNIRS is capable of penetrating several centimeters through brain tissue to capture human brain structures accurately (Min et al., 2017; Pinti et al., 2020), we chose a relatively narrow spectral range (540–650 nm) because the penetration is more superficial (~0.39 mm due to higher scattering) and our chosen range contains not only multiple isobestic points in the hemoglobin absorption spectra for oxygenated vs. de-oxygenated around 550 nm, but also a point around 640 nm where absorption by oxyhemoglobin is 10-fold lower than absorption by deoxyhemoglobin. Our method offers measurement in a banana-shaped volume between a source fiber that illuminates the brain surface and a radially-displaced detector fiber (see Supplementary Figure 1). As a result, visible light spectroscopy can provide a more precise measurement for small mouse brains than fNIRS. With deeper implantation of the probe fibers, cerebrovascular measurements of deep brain regions, such as the hypothalamus or amygdala, in response to complex behavioral tasks will be possible with our approach.

The ability to study cerebrovascular reactivity in freely moving animals without anesthesia could greatly contribute to a more accurate understanding of oxygen needs, minimize stress, and enable the analysis of cerebrovascular changes during the behavior of animals in various animal models of neurological disorders. In order to overcome these limitations, we developed a device and method for measuring cerebral blood volume fraction (*CBVF*), the percent volume of blood in the measured volume of brain tissue, and oxygen saturation (*S_O2_*), the ratio of oxygenated hemoglobin to total hemoglobin, in freely moving animals using fiberoptic spectroscopy. Cerebrovascular tone and cerebral blood oxygen level are critical parameters of cerebral circulation that can be measured with *CBVF* and *S_O2_* using visible-light diffuse-reflectance spectroscopy. These measurements depend on the balance of blood flow and oxygen consumption by the tissue, making them attractive candidates to monitor cerebral circulation as used in functional magnetic resonance imaging (fMRI) and functional near-infrared spectroscopy (fNIRS). The measurement of *CBVF* is closely linked to cerebrovascular reactivity in the brain. When exposed to hypercapnic conditions, blood vessels in the brain dilate to match the tissue’s increased demand for blood, a process known as CVR to CO_2_. One way to investigate CVR is by measuring the changes in CBF or cerebral blood volume (CBV) induced by vasodilation (Grubb et al., 1974; Sleight et al., 2021). In this study, we measured the change of *CBVF* and *S_O2_* as a percentage in tissue during hypercapnia, which can indicate cerebrovascular reactivity events. Furthermore, we examined *CBVF* and *S_O2_* during rest and running of animals using the rotarod test, as CVR has been linked to physical exercise (Braz and Fisher, 2016; Miller and Mitra, 2017; Steventon et al., 2020).

## 2. Materials and methods

### 2.1. System and computational method of fiberoptic measurement

A white light source (HL-2000-HP, Ocean Optics) illuminated the cerebral cortex through an illumination fiber bundle of 8 fibers (FG105UCA, Thorlabs) in 7-around-1 mated to a solid core fiber in the tip (FT400UMT, Thorlabs), or a solid core plastic polymer fiber (DIY-Fiber-500, Prizmatix) mated to the same fiber material in the tip, while an identical detection fiber tip and bundle directed the measured diffuse reflectance to a spectrometer (HDX, Ocean Optics). Our system was controlled through Matlab (Mathworks) software by a laptop computer running Windows 10. Spectra were fit for real-time readout of *CBVF* and *S_O2_* on the perioperative laptop during experimentation. The graphic user interface is provided in Supplementary material. The sampling rate of the spectroscopic measurement (generally ~1 Hz) is set and determined by the spectrometer integration time, which is automatically set over iterative measurements during spectroscopy to achieve a signal that is in the high, but not saturated region of between 70% and 90% of the spectrometers’ specified saturation level.

### 2.2. Spectroscopy

Calibration spectra *C*(*λ*) were acquired using measurements of a 99% reflectance standard (AS-011XX-X60, Labsphere, North Sutton, NH) while holding the fiber probe 3 cm from the standard. The dark noise of the spectrometer was also measured (*dark*) with the illumination lamp off. Calibrated spectral measurements *M*(***λ***) of tissue were calculated from the raw tissue spectral measurements *R*(***λ***) and the calibration measurements *C*(***λ***) according to Equation 1. Measured spectra depended on the spectra of the light source and the detector sensitivity but these factors were canceled by the normalization:


(1) $$M\left(\lambda \right)=\frac{R\left(\lambda \right)-dark}{C\left(\lambda \right)-dark}$$

A theoretically predicted spectral measurement *M_p_(**λ**)* was created by multiplying the predicted reflectance, *R_p_(**λ**),* by a scaling factor K: *M_p_(**λ**)* = *KR_p_(**λ**)* as in equation 12, below. Least squares minimization between *M_p_(**λ**)* and *M(**λ**)* specified the endpoint metrics *S_O2_* and *CBVF*. Equations 3–13 implement diffusion theory (Egan, 1979; Groenhuis et al., 1983a,b; Farrell et al., 1992) to calculate R_p_*(**λ**)* as adopted from our previous esophagus studies (Gareau et al., 2010; Pham et al., 2011).

Equations 3–12 are implemented in the simulated diffuse reflectance spectrum in the supplementary Matlab spectroscopy code (GUI.m) and the key diffusion parameters are defined in the referenced literature, such as the internal reflection coefficient *A* for light totally internally reflecting within the media at the media boundary, the equivalent point source depth *z_0_**(λ)**,* diffusion constant *D(**λ**)* and the effective attenuation coefficient $\mu_{eff}\left(\lambda \right).$ Diffusion theory was considered valid since the 2.5 mm source-detector separation on our probe was large compared to the transport scattering mean free path (*MFP’* = 0.39 mm), calculated using scattering spectra (Supplementary Figures 2A,B) and for the least scattering wavelength in our measurement (650 nm). The reduced mean free path (*MFP’*) is effectively the probing depth of optical penetration.


(2) MFP′=1(μsTissue(600nm)∗(1−gTissue(600nm)))=0.39mm

where “Tissue” denotes the approximate mixed brain tissue type to be 1/3 white matter and 2/3 gray matter as in Equations 14, 15, below.


(3) $$r_{i}=0.6681+0.0636n+\frac{0.7099}{n}-\frac{1.4399}{n^{2}}$$

An internal reflection parameter (*A*) that accounts for the refractive index mismatch at the boundary between the tissue and the external medium is calculated based on (*r_i_*).


(4) $$A=\frac{1+r_{i}}{1-r_{i}}$$

Diffuse reflectance (light escaping the tissue surface) emanates as if from an equivalent point source depth (*z_0_*) within the tissue.


(5) $$z_{0}\left(\lambda \right)=\frac{1}{\mu_{a}\left(\lambda \right)+\mu_{s}{}^{\prime }\left(\lambda \right)}$$

The diffusion constant (*D*) is calculated:


(6) $$D\left(\lambda \right)=\frac{z_{0}\left(\lambda \right)}{3}$$

The effective attenuation coefficient is derived from the diffusion constant and the absorption.


(7) $$\mu_{eff}\left(\lambda \right)=\frac{1}{\sqrt{\frac{D\left(\lambda \right)}{\mu_{a}\left(\lambda \right)}}}$$

The Greens function describing fluence at a point separated from the source fiber by a distance (*r*) uses two geometrical factors (*r_1_*) and (*r_2_*).


(8) $$r_{1}\left(\lambda \right)=\sqrt{z_{0}{\left(\lambda \right)}^{2}+r^{2}}$$

(9) $$r_{2}\left(\lambda \right)=\sqrt{{\left(z_{0}+4AD\left(\lambda \right)\right)}^{2}+r^{2}}$$

Two other variables— c(λ)andd(λ) —are needed to calculate the diffuse reflectance.


(10) $$c\left(\lambda \right)=z_{0}\left(\lambda \right)\times \left(\mu_{eff}\left(\lambda \right)+\frac{1}{r_{1}\left(\lambda \right)}\right)\times \frac{e^{\frac{-r_{1}\left(\lambda \right)}{\Delta \left(\lambda \right)}}}{r_{1}{\left(\lambda \right)}^{2}}$$

(11) $$d\left(\lambda \right)=\left(z_{0}\left(\lambda \right)+4AD\left(\lambda \right)\right)\times \left(\mu_{eff}\left(\lambda \right)+\frac{1}{r_{2}\left(\lambda \right)}\right)\times \frac{e^{\frac{-r_{2}\left(\lambda \right)}{\Delta \left(\lambda \right)}}}{r_{2}{\left(\lambda \right)}^{2}}$$

The predicted fiberoptic measurement (*M_p_*) based on diffusion theory is a constant (K) multiplied by the predicted diffuse reflectance *R_p_* (λ) .


(12) $$M_{P}\left(\lambda \right)=KR_{P}\left(\lambda \right)=K\frac{c\left(\lambda \right)+d\left(\lambda \right)}{4\pi }$$

The fiber separation (distance between the source fiber tip and the detector fiber tip) was *r* ≈ 0.25 cm, and the refractive index mismatch between glass (n_glass_ = 1.52), or plastic polymer fiber (n_polymer_ = 1.49) and tissue (n_tissue_ = 1.4) was n = n_glass_/n_tissue_ = 1.09 or n = n_polymer_/n_tissue_ = 1.06 *μ_a_(**λ**)* and *μ_s_’(**λ**)* were the absorption and reduced scattering coefficients of the tissue, respectively. The spectral absorption coefficient, *μ_a_(**λ**)*, was calculated as a weighted sum of the absorption spectra of oxygenated whole blood [*μ_a_oxy_(**λ**)*], deoxygenated whole blood [*μ_a_deoxy_(**λ**)*], and water [*μ_a_water_(**λ**)*], according to Equation 13:


(13) $$\mu_{a}\left(\lambda \right)=CBVF\times \left[\begin{array}{l} S_{O2}\times \mu_{a_{oxy}}\left(\lambda \right)+\\ \left(1-S_{O2}\right)\times \mu_{a_{deoxy}}\left(\lambda \right) \end{array}\right]+W\times \mu_{a_{water}}\left(\lambda \right)\;$$

### 2.3. Calculation of fitting parameters to quantify *S_O2_* and *CBVF*

Spectra were analyzed in the range 540 to 650 nm by least-squares fitting. The rationale for this wavelength range is that the optical penetration is restricted to the superficial brain layers including the cortex and that the range includes multiple isosbestic points of the hemoglobin absorption curve so it is rich in information content regarding hemoglobin saturation. Regarding the superficial penetration, our visible light at approximately 600 nm penetrated to a depth of the *MFP*, which is 0.39 mm (Equation 2) whereas optical scattering properties of mouse brain tissue at the 950 nm near infrared wavelength are less diffusing, with a deeper sampling (Min et al., 2017).

The difference between *M* (Eq. 1) and *M_p_* = KR_p_ (see Eq. 12) was minimized by adjusting *M_p_* using a multidimensional unconstrained nonlinear minimization method (Nelder–Mead), the fminsearch() in Matlab. Fitting adjusted 3 variables: *CBVF, S*_*O2*,_ and *K* where *CBVF* = 1 specifies 150 g/L hemoglobin. *W,* the fractional tissue water content, was fixed at an assumed value of 0.78, which has been reported for the mouse cortex (Nakamura et al., 2004). Absorption due to water was minimal. For example, at 650 nm, low *CBVF* = 0.01 and *S_O2_* = 0.5, the absorption coefficient in the tissue due to the presence of blood is *μ*_*a*_blood_ = *CBVF*×*S_O2_* × *μ*_*a*_oxy_ + *CBVF*×(1-*S_O2_*) × *μ*_*a*_deoxy_ = 0.1103 cm^−1^. *μ_a_water_* = 0.0025 cm^−1^, which is 44-fold lower than *μ*_*a*_blood_.

The absorption of the tissue due to blood was specified by the fitting described above while the scattering optical properties (scattering coefficient *μ_s_(**λ**)* [cm^−1^] and scattering anisotropy *g(**λ**)* [−]), which were constant across all experiments, were spectral functions *(**λ**)* fit to the previous measurements of fresh human brain tissue (Yaroslavsky et al., 2002). Thus to specify tissue scattering properties, *μ_s_(**λ**)* and *g(**λ**)* were used as interpolated functions of wavelength (Equations 14, 15) that, in combination yielded the reduced scattering coefficient, *μ_s_’(λ) = μ_s_(λ)* × (1 − g*(λ)*) used in Equation 5. These optical properties agree reasonably with the literature (Yaroslavsky et al., 2002; Jacques, 2013), such as our previous data (Yaroslavsky et al., 2002) that can be fit for the reduced scattering coefficients of gray and white matter as shown in Supplementary Figure 2C.

### 2.4. Brain optical scattering properties

Supplementary Figure 2 shows the brain scattering properties specified by the spectral data from Yaroslavsky et al., 2002 (Yaroslavsky et al., 2002). Supplementary Figures 2A,B show the spectral behavior of the scattering coefficient (*μ_s_*) and the anisotropy of scattering (g). The combination of *μ_s_* and g yields the reduced scattering coefficient [*μ_s_*’ = *μ_s_*(1 − g)] shown in Supplementary Figure 2C for white and gray matter. Scattering properties of whole tissue were set as a weighted-sum based on a 1:2 ratio of white to gray matter, as reported (Zhang and Sejnowski, 2000) for the mouse cortex.


(14) $$g_{Tissue}\left(\lambda \right)=g_{WhiteMatter}\left(\lambda \right)\times \frac{1}{3}+g_{GrayMatter}\left(\lambda \right)\times \frac{2}{3}$$

(15) $$\mu_{s_{Tissue}}\left(\lambda \right)=\mu_{s_{WhiteMatter}}\left(\lambda \right)\times \frac{1}{3}+\mu_{s_{GrayMatter}}\left(\lambda \right)\times \frac{2}{3}$$

The *μ_s_*’(***λ***) spectrum was assumed when using least-square fitting of the data to specify *K*, *CBVF,* and *S_O2_*. After spectral fitting, the values for *CBVF* and *S_O2_* were output as a function of time over time periods where spectroscopic measurements were made on mice under various experimental conditions. These optical properties were also used to model the photon transport in the mouse brain.

### 2.5. Fabrication of fiberoptic probe with surgically implantable, magnetically-coupled tip

**Figure 1:**
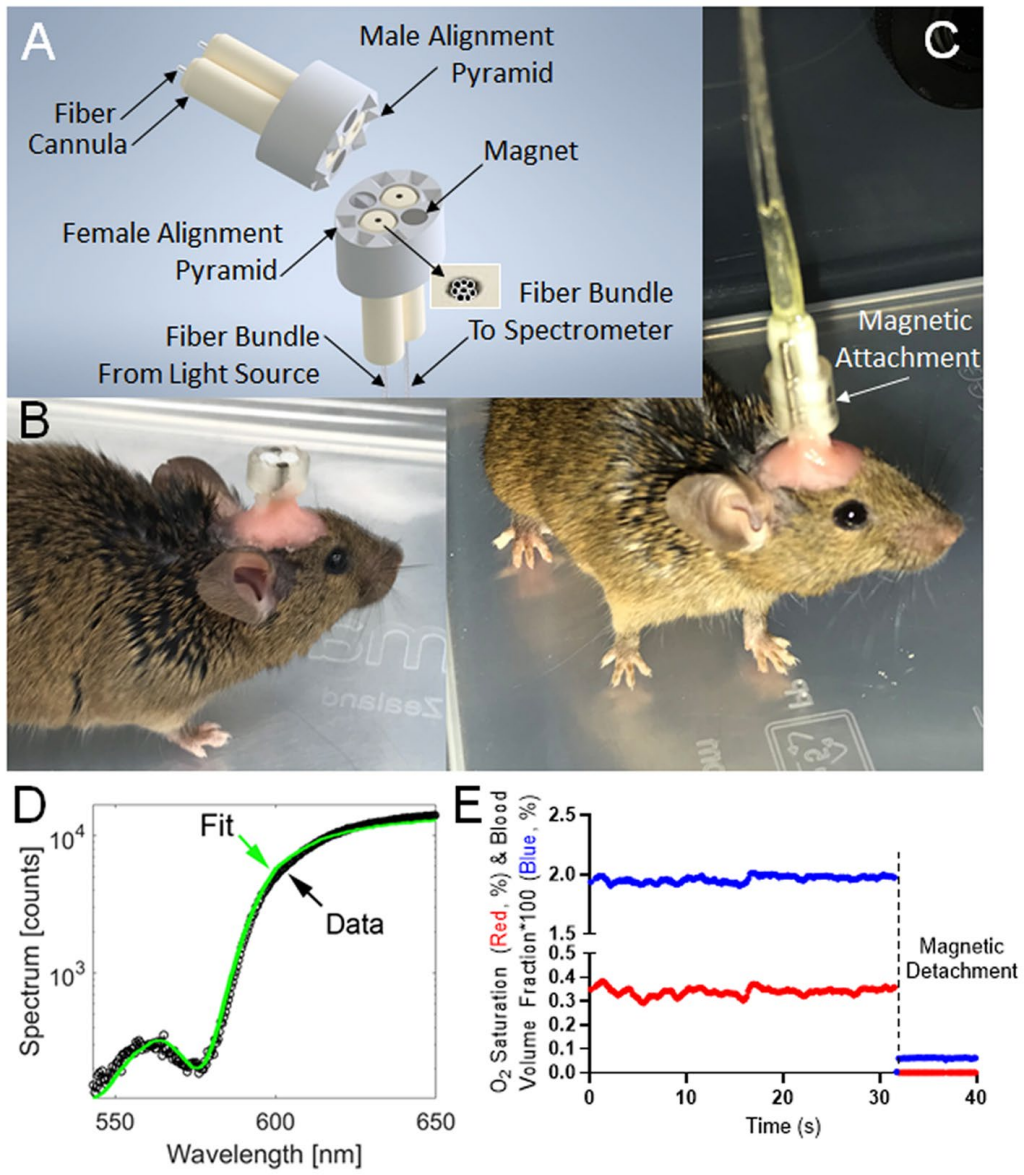
Instrumentation and typical readout for Fiberoptic cerebral oximetry in freely moving mice. **(A)** 3D model of the magnetic-coupled probe. Six male/female mating pyramid pairs secure the detachable, implantable brain probe into a linking connector with mating female pyramid components to enable fiberoptic alignment. **(B)** Surgically-implanted brain probe. **(C)** Brain probe connected to a spectrometer. **(D)** Spectral fitting in <0.25 s yielding instantaneous measurements of the percentages of *CBVF* and *S_O2_* in the 540 nm to 650 nm range chosen to contain 3 isosbestic points of hemoglobin spectral absorption alongside an anchoring wavelength (650 nm) of differential absorption. **(E)** Typical time-course measurement over resting behavior and decoupling event corresponding to magnetic probe detachment. The graph shows cerebral blood volume fraction (blue) times 100 such that a y-axis value of 1 means 1% blood volume fraction and the oxygen saturation fraction (red), which is the fraction of hemoglobin molecules bound to oxygen within the cerebral probed volume.

The connection between the mouse brain and the spectrometer consisted of an implanted brain probe (~1 cm fiber) and a spectrometer connector (~2 m fiber). The connector (Figure 1A) is fiber-optically coupled to the illumination source and the spectrometer and opto-magnetically coupled to the brain probe, which is fiber-optically coupled to the brain. Plastic connectors were 3D-printed using a 3D Systems ProJet printer and VisiJet M3 Crystal material. Mating surfaces were 3D-printed facing upward (opposite the print bed) to minimize warp and maximize surface flatness. The plastic connector design was optimized to align and hold (press fit) two ceramic ferrule cannulas (CF440-10, Thor Labs, Newton NJ) and two cylinder magnets (D12-N52, K&J Magnetics, Plumsteadville PA).

During assembly, the connector’s flat surface was pressed against the glass, and the ferrules and magnets were inserted and also pressed against the glass for alignment (friction fit). The male side connector was fabricated using a similar process of press-fitting the cannulae and magnets, but the assembly was pressed against an alignment tool (see Supplemental material), instead of the flat glass plate. The connector was then mated to its probe with magnetic attachment, and pre-polished fibers (FT400UMT, Thor Labs, Newton NJ) were inserted into the ferrules and affixed using UV-cure adhesive (#68, Norland Products, Cranbury NJ). After drying, the protruding fibers were clipped and polished to a length of 0.5 mm protruding from the cannulas using a custom polishing tool set. The CAD file of a polishing toolset is available in the repository at Rutgers University Libraries.^1^

In order to maintain alignment, so that the fibers in the brain probe and spectrometer connector mated precisely, the brain probe surface was printed with 6 small 4-sided pyramids while the connector surface was printed with complementary mating holes. This design configuration enabled the secure mating of the two connecting parts during measurements. Finished fiber probes were visually inspected under a 20X stereomicroscope for polish level and analyzed through a series of signal and noise tests using the spectroscopy probe in a dark room and the light source targeted at human skin.

The quantitative performance parameter for magnetic coupling was the amount of white light that leaked directly from the source fiber bundle to the detector fiber bundle. This amount defined the noise and was measured with the surgically-implantable tip magnetically coupled and the illumination light going from it toward the Vantablack target in a dark room. The quantitative spectrometer readout, in counts, was read out (*R1*) at the 650 nm wavelength. A second count readout (*R2*) was measured with the probe contacting human skin. The ventral skin surface is less pigmented and the easily accessible, finger pad has little unwanted influence of melanin. The signal-to-noise ratio was then [*R1* – *R2*]/*R1* and the manufacturing quality control threshold was set as a 60-1 signal-to-noise ratio so that, as a first-order approximation, an extra 1/60th of an optical measurement of the brain consisting of leaked white light would erroneously decrease the absolute *CBVF* no more than 100%/60 = 1.6%.

### 2.6. Analysis of cerebrovascular reactivity in freely moving mice

5-month-old C57BL/6 mice (The Jackson Laboratory) were used to measure basal cerebrovascular dynamic properties of the cortex while freely moving. The AD model mice studied were 13-month-old Tg6799, also called 5xFAD (Jackson Laboratory) double transgenic mice overexpressing both human amyloid precursor protein (APP) gene with KM670/671/NL, V717I, and I716V mutations and human presenilin 1 (PSEN1) harboring M146L and L286V mutations under the Thy1 promoter (Oakley et al., 2006). Wildtype (WT) littermates were used as controls. Only male mice were used in experiments. All mice were maintained on a 12-h light/dark cycle and given *ad lib* access to irradiated mouse chow and water for the duration of the experiment. All experiments were done according to policies on the care and use of laboratory animals of the Ethical Guidelines for Treatment of Laboratory Animals of the NIH. Relevant protocols were approved by the Rockefeller and Rutgers Institutional Animal Care and Use Committee (IACUC).

After anesthesia with isoflurane mixed with pure oxygen (3% isoflurane and 1 L/min oxygen for induction and 1.5% isoflurane and 1 L/min oxygen for maintenance), mice were mounted on a stereotactic frame. After the removal of hair over the scalp, the scalp was cleaned with sterile alcohol and Betadine three times. We performed a minimal-sized midline scalp incision (~5 mm). We then drilled two small holes in the skull using a 0.7 mm diameter stainless steel micro drill burr (Fine Science Tools). The coordinates of the two holes in reference to Bregma were anterior = −1 mm, lateral = 1.5 mm and anterior = −3.5 mm, lateral = 1.5 mm. Brain probes were fitted to the holes and immobilized using dental cement. The skin incision was sutured and animals were monitored throughout the recovery period. A week after surgery, mice were placed into a chamber (22.8 cm × 20.3 cm × 15 cm) and we measured cerebral perfusion in the freely moving state.

For the free moving recording study, we allowed the animal to freely move in an open field and measured *CBVF* and *S_O2_* while video recording the animal. After the measurement, we classified the animal activity seen in the video recording as grooming, resting, or walking activity. In order to assert more control over animal behavior during fiberoptic measurements, we measured *CBVF* and *S_O2_* during an accelerating rotating rod (Rotarod) behavior test. For the Rotarod behavior test, we allowed the animal to rest on the Rotarod device for 60 s and then started the device for 180 s. The initial speed during the running period was set to 1 rpm and ramped up to 10 rpm over 60 s, staying at the top speed for another 120 s. After the 180 s running period, the animal was allowed to rest for 120 s for a total measurement period of 360 s. For the hypercapnia study, we injected 10%CO_2_/10% O_2_/80%N_2_ mixture into the induction chamber and measured changes in *CBVF* and *S_O2_* during and after hypercapnia.

## 3. Results

### 3.1. Development of fiberoptic cerebral oximetry in freely moving mice

To investigate cerebrovascular changes in freely moving mice, we developed a connection between the mouse brain and the spectrometer consisting of an implanted brain probe (~1 cm fiber) and a spectrometer connector (~2 m fiber). The device (Figure 1A) fiber-optically coupled the brain to the light source and the spectrometer when magnetically coupled but could be detached so that the probe could be surgically implanted before surgical recovery and subsequent measurement during normal behavior (Figures 1B,C). After recovery and attachment, spectral measurements were analyzed by least-square fitting to light transport theory (Figure 1D) to specify the instantaneous *S_O2_* and *CBVF* (Figure 1E). These values were significantly changed when decoupling event corresponding to magnetic probe detachment (Figure 1E).

**Figure 2:**
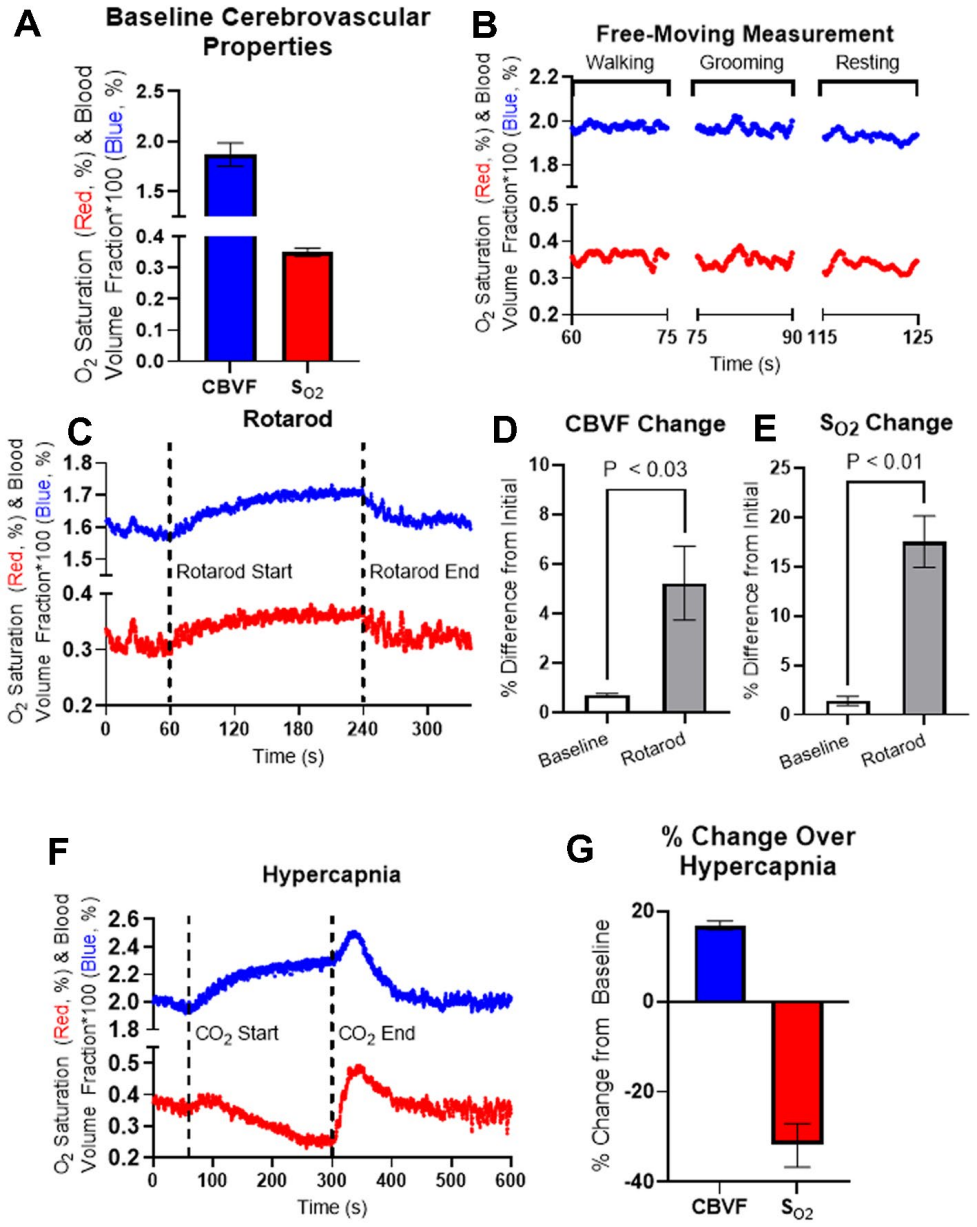
Fiberoptic measurement of 5-month-old C57BL/6 mice during the behavior and hypercapnia. **(A)** Averaged baseline *CBVF* and *S_O2_* measurements of 5-month-old C57BL/6 mice (*n* = 4). **(B)** Representation for cerebrovascular changes during activity classified as either walking, grooming, or resting during a measurement of a freely moving, 5-month-old C57BL/6 mouse. **(C)** Cerebrovascular responses in 5-month-old mice were obtained using fiberoptic spectroscopy during Rotarod experiments. Running behavior was induced by the Rotarod device at 60 s and stopped at 240 s as the animal was allowed to rest until 360 s. **(D,E)** The average values of *CBVF* and *S_O2_* during 60 s of basal measurement as well as the maximum values of *CBVF* and *S_O2_* over the running period of the experiment were measured as a percent change from the average *CBVF* and *S_O2_* values of the first 10 s measurement. **(D)** The percent change of the maximum *CBVF* value over the running period was found to be significantly higher than basal measurement (*p* < 0.03, *n* = 4). **(E)** The percent change of the maximum *S_O2_* value over the running period was also found to be significantly higher than basal measurement (*p* < 0.01, *n* = 4). **(F)** Cerebrovascular responses in 5-month-old freely moving mice were obtained using fiberoptic spectroscopy during hypercapnia. Hypercapnia was induced at 60 s by increasing CO_2_ concentration up to 10% and was kept until releasing CO_2_ at 300 s. **(G)** Maximum *CBVF* and minimum *S_O2_* over the course of hypercapnia were measured as a percent change from the baseline averages. Bar graphs represent mean ± s.e.m.; *n* = 4; statistical analyses were performed using two tailed unpaired *t*-test.

Fiber spectroscopic measurement of *CBVF* and *S_O2_* in mixed arterial and venous blood of the cortex of freely moving 5-month-old C57BL/6 mice yielded values of ~1.9% and ~35%, respectively (Figure 2A). We were interested in the effect of animal behavior on measurements of *CBVF* and *S_O2_* from the fiberoptic device. To investigate these effects, we measured *CBVF* and *S_O2_* in our free moving experiment and tracked the time that each animal was exhibiting grooming, resting, and walking behavior over the course of the measurements (Figure 2B). We found that there was no significant difference in average *CBVF* and *S_O2_* values between the three classifications of behavior.

In order to have more control over animal behavior exhibited over the course of fiberoptic measurement, we measured *CBVF* and *S_O2_* in our accelerating rotarod experiment to control running and resting behavior in the mice. During the accelerating rotarod experiment, *CBVF* and *S_O2_* measurements were taken over 360 s where the animal rested for the first 60 s, ran for the next 180 s, and then rested again for the final 120 s (Figure 2C). In order to understand the effects of running on *CBVF* and *S_O2_*, the average baseline *CBVF* and *S_O2_* values over the first 60 s of rest and the maximum *CBVF* and *S_O2_* values over the course of the running period were projected as the percent of change as compared to the average *CBVF* and *S_O2_* values of the first 10 s of the beginning of the test. We found a significant increase in the percent change of the maximum *CBVF* (5.2 ± 1.5%) and *S_O2_* (17.5% ± 2.6%) values over the course of the running period as compared to the percent change of the average *CBVF* and *S_O2_* values of the baseline (Figures 2D,E). Our results suggest that minor behavior changes such as walking and grooming do not significantly affect the cerebrovascular properties measured by our device, but constant exercise such as running on the rotarod significantly increase *CBVF* and *S_O2_*.

To investigate the change in *CBVF* and *S_O2_* of the cortex during hypercapnia in the mouse cortex, we placed mice in an insulated chamber and increased CO_2_ concentration up to 10%. When hypercapnia was induced after 60 s of baseline measurement by increasing CO_2_ concentration, we found an increase in *CBVF* (16.9 ± 1.0%), which is a typical hyperaemic response to increased CO_2_ through vasodilation. *S_O2_* decreased (−31.8% ± 2.4%) slowly during hypercapnia but normalized rapidly after stopping CO_2_ injection and releasing CO_2_ at 300 s, while *CBVF* increased before returning to a basal level (Figures 2F,G).

### 3.2. Impaired cerebrovascular reactivity in freely moving AD mice

**Figure 3:**
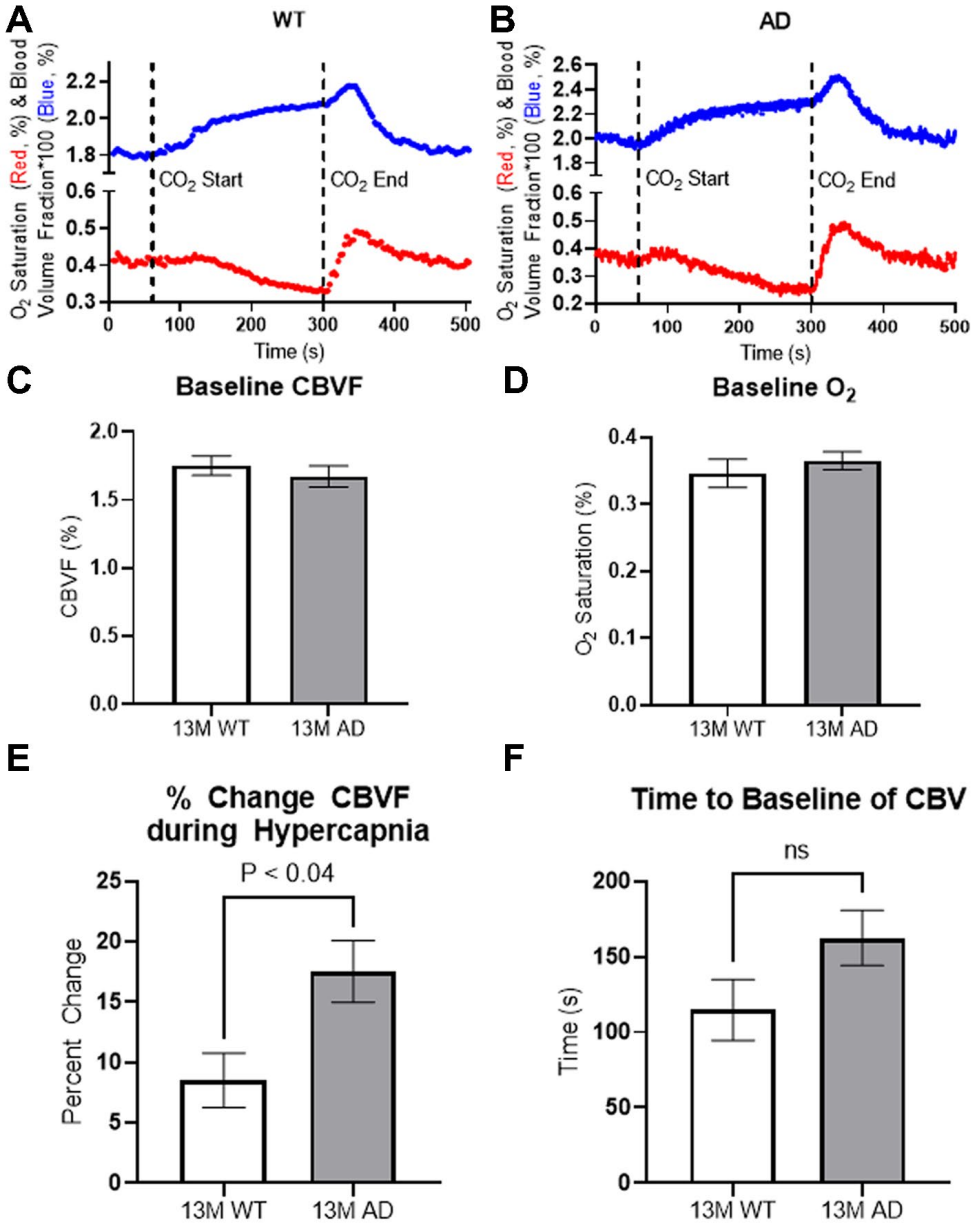
*In vivo* spectroscopy results. **(A,B)** Representation for cortical vascular reactivity of WT littermate **(A)** and Tg6799 13-month-old mice **(B)** during hypercapnia was analyzed using fiberoptic spectroscopy. Hypercapnia was induced at 60 s by increasing CO_2_ concentration up to 10% and was kept until releasing CO2 at 300 s. **(C,D)** 13-month-old Tg6799 mice and WT littermates showed similar levels of basal oxygen saturation **(D)** and cerebral blood volume fraction (*CBVF*) **(C)** in the cortex. **(E)** The increase of *CBVF* during the hypercapnia was significantly higher in Tg6799 mice compared to WT littermate (*p* < 0.04, *n* = 4 AD, 4 WT). **(F)** There is a tendency of delayed time to the baseline of *CBVF* after hypercapnia in Tg6799 mice, but it is not statistically significant (*n* = 4 AD, 4 WT). Bar graphs represent mean ± s.e.m.; *n* = 4 AD, 4 WT; analyzed by two tailed unpaired *t*-test.

To investigate the cerebrovascular properties of AD (Tg6799) mice, we measured the baseline of *CBVF* and *S_O2_* of the cortex of freely moving 13-month-old Tg6799 mice and wild-type (WT) littermates and also analyzed the difference in vascular reactivity between Tg6799 mice and WT littermates during and after hypercapnia using fiberoptic spectroscopy (Figures 3A,B). While there was no difference in the baseline of *CBVF* and *S_O2_* between the two groups (Figures 3C,D), the increase of *CBVF* of the cortex of AD mice during hypercapnia was significantly higher than that of WT littermates (Figure 3E). In addition, although not statistically significant, there was a strong tendency of delayed recovery time to the baseline of *CBVF* of AD mice in comparison to WT littermates (Figure 3F).

To assess longitudinal measurement and repeatability, we performed several measurements over time in the same animal and found that repeated measurements were stable between 1 and 3 weeks after the brain probe implantation surgery. Supplementary Figure 3 shows repeated measurements from one 5-month-old animal (Supplementary Figures 3A,B). Fiberoptic measurements with hypercapnia-induced vasodilation were also taken at 1 and 3 weeks postsurgery, and show similar responses to hypercapnia conditions between the two time points (Supplementary Figures 3C,D). These results suggest that the longitudinal measurement of basal level cerebrovascular properties and hypercapnia-induced cerebrovascular change are stable.

## 4. Discussion

Fiberoptic spectroscopy can assess the *S_O2_* and the *CBVF* in the brains of freely moving mice when implemented with a magnetic coupling tip. The present study showed that our fiberoptic method detected changes in freely moving mice and measured cerebrovascular impairment in a mouse model of AD. Previous studies on *S_O2_* in the cortex of anesthetized rodents (Vovenko, 1999; Sakadžić et al., 2010) have demonstrated that *S_O2_* or oxygen partial pressure (*pO_2_*) exhibits high heterogeneity based on the types of blood vessels, such as arterioles, venules, and capillaries. Moreover, Sakadzic et al. observed a decline in vascular *pO_2_* with an increase in cortical depth. Considering that our device measured mixed arterial and venous blood of the cortex at a depth of 0.3–0.7 mm (Supplementary Figure 1), the 30%–40% *S_O2_* measurement in the cerebral blood of mice is comparable to that of previous studies. The average *CBVF* of 5-month-old C57BL/6 mice was ~1.9% (Figure 2A) and *CBVF* of 13-month-old Tg6799 (C57BL/6 background) was ~1.7% (Figure 3C). This range from 1.7% to 1.9% in our results is slightly less than the range reported in other literature: 2.1% reported by Synchrotron Radiation Quantitative Computed Tomography in the parietal cortex (Adam et al., 2003), 2.03%–3.14% reported by MRI in the cortex (Dunn et al., 2004; Huang et al., 2013; Han et al., 2015), 2.0%–2.4% reported by multiphoton laser scanning microscopy and in the capillary-rich cerebral cortex (Verant et al., 2007). One possible reason is that we measured *CBVF* of 5- and 13-month-old mice, instead of young rodents (4–8 weeks old), which other studies mainly used (Dunn et al., 2004; Verant et al., 2007; Huang et al., 2013; Han et al., 2015). Another possible reason is that large blood vessels on the surface of the brain such as meningeal vessels were excluded from our assessment because our fibers were implanted ~0.3 mm below the skull, which can be seen in our immunohistochemical staining of a surgery mouse brain with antibodies against collagen and DAPI staining (Supplementary Figure 1B).

Several previous studies have explored the development of devices for monitoring cerebrovascular properties in freely-moving animals, including head-mounted microscope devices (Sigal et al., 2016; Senarathna et al., 2019). While the miniature multi-contrast microscope developed by the Pathak group provides high spatio-temporal resolution of cerebral blood flow (CBF) and cerebral blood volume (CBV) combined with several advanced technologies in freely moving mice (Senarathna et al., 2019), our system offers greater freedom of movement for the animals (Supplementary Video 1), enabling us to measure *S_O2_* and *CBVF* during a wider range of behavior tests, such as the rotarod, and objective recognition tests. Furthermore, our device provides specific *S_O2_* and *CBVF* values for targeted brain regions, enabling investigation of age-dependent changes and differences between animal models. In contrast to head-fixed mouse approaches, our system permits cerebrovascular measurements of deeper brain regions, such as the thalamus or striatum, through deeper implantation of the probe fibers. However, a current limitation of our device is its lower spatial resolution, which will need to be improved in future studies.

Tg6799 mice start to develop amyloid plaque deposits by 2 months of age (Oakley et al., 2006) and show a substantial amount of amyloid plaque deposits and cerebral amyloid angiopathy in their cortex (Ahn et al., 2014). In addition, recent dynamic susceptibility contrast-enhanced (DSC) MRI analysis showed increased vasodilation in 12-month-old Tg6799 mice compared to littermate control, in response to contrast injection (Tataryn et al., 2021). Contrast injection to the lateral tail vein increases systemic blood pressure and might induce a similar vascular effect from hypercapnia. This result is quite similar to the current study showing increased vascular reactivity in Tg6799 mice. The study using another AD mouse model, J20-hAPP, also showed increased vasodilation in the brains of AD mice under hyperoxia compared to WT controls (Shabir et al., 2020). Although cerebral hypoperfusion is typically observed in advanced AD patients (Du et al., 2006; Austin et al., 2011), an increase in cerebral perfusion was also seen in some cases of early AD patients (Alsop et al., 2008; Luckhaus et al., 2008; Ding et al., 2014). Integrating several studies of cerebral blood flow (CBF) in AD patients suggests that there is cerebral hyperperfusion in early disease followed by hypoperfusion later in the disease (Chen et al., 2011; Østergaard et al., 2013). In AD patients, as the disease progresses, severe neuronal cell death and brain atrophy have been widely reported (Raskin et al., 2015; Weiner et al., 2015). However, our AD mouse model (Tg6799) showed very minor neuronal cell death in limited brain areas and no brain atrophy. Therefore, Tg6799 may represent the early stage of the disease, and hyperperfusion of this mouse model could be relevant to early AD patients.

Though reliable *S_O2_* and *CBVF* values over longitudinal measurements have been shown here, another limitation is the lack of validation of absolute estimates of these parameters across animal models, and results thus far may be subject to estimation error. Mechanically, our device allows natural animal movement because of the flexibility of our fiber bundle and a video of an exemplary animal’s free movement is provided as Supplementary Video. When animal movement becomes so severe that continued connectivity of the device would become dangerous to the animal, our magnetic detachment clean break-away protects the animal and therefore measurement integrity of future measurements. Figure 1E shows the distinction between resting fit measurements and the fitting result when the magnetic attachment (which assures fit quality) has been broken. This fiberoptic method may improve understanding of the mechanisms of impaired vascular reactivity in AD. Our vasculature and hemodynamic measurements raise the question as to whether the observed phenomena are consequences of the pathology or more fundamental changes that are required for disease progression. We hope to explore these temporal measurements in future work, potentially combining *CBVF* and *S_O2_* with time-differential mathematics to access perfusion and oxygen consumption parameters.

## Data availability statement

The datasets presented in this study can be found in online repositories. The names of the repository/repositories and accession number(s) can be found at: https://scholarship.libraries.rutgers.edu/esploro/outputs/technicalDocumentation/Code-data-and-materials-for-fiberoptic/991031663984304646?institution=01RUT_INST.

## Ethics statement

The animal study was reviewed and approved by Rutgers University and Rockefeller University.

## Author contributions

DG, NR, AC, MB, JZ, and HA performed the experiments. DG, NR, AC, AY, SJ, and HA analyzed the data and wrote the manuscript. DG, MS, SS, JK, and HA designed the study. DG and HA participated in the interpretation of data and manuscript preparation. All authors contributed to the article and approved the submitted version.

## Funding

Research reported in this publication was supported by the National Institute on Aging of the National Institutes of Health under Award Number RF1AG078245 (HA), by the National Institute of Neurological Disorders And Stroke of the National Institutes of Health under Award Number R01NS104386 (HA), and the Robertson Therapeutic Development Fund (HA). The content is solely the responsibility of the authors and does not necessarily represent the official views of the National Institutes of Health.

## Conflict of interest

The authors declare that the research was conducted in the absence of any commercial or financial relationships that could be construed as a potential conflict of interest.

## Publisher’s note

All claims expressed in this article are solely those of the authors and do not necessarily represent those of their affiliated organizations, or those of the publisher, the editors and the reviewers. Any product that may be evaluated in this article, or claim that may be made by its manufacturer, is not guaranteed or endorsed by the publisher.

## Footnotes

^1^https://scholarship.libraries.rutgers.edu/esploro/outputs/technicalDocumentation/Code-data-and-materials-for-fiberoptic/991031663984304646?institution=01RUT_INST
